# Innovative Models for High-Risk Patients Use Care Coordination and Palliative Supports to Reduce End-of-life Utilization and Spending

**DOI:** 10.1093/geroni/igx021

**Published:** 2017-11-20

**Authors:** Sarah Ruiz, Lynne Page Snyder, Katherine Giuriceo, Joanne Lynn, Erin Ewald, Brittany Branand, Shriram Parashuram, Sai Loganathan, Tyler Bysshe

**Affiliations:** 1 National Institute on Disability, Independent Living, and Rehabilitation Research, Administration for Community Living, Washington, District of Columbia; 2 NORC at the University of Chicago, Health Care Department, Bethesda, Maryland; 3 Center for Medicare and Medicaid Innovation (CMMI), Centers for Medicare & Medicaid Services, Baltimore, Maryland; 4 Center for Elder Care and Advanced Illness, Altarum Institute, Washington, District of Columbia

**Keywords:** Advance care planning, Alternative payment models, Care transitions, Disease management, Evidence-based programs, Multiple chronic conditions, Quality improvement

## Abstract

**Background and Objectives:**

Care coordination and palliative care supports are associated with reduced anxiety, fewer hospital admissions, and improved quality of life for patients and their families. Early palliative care can result in savings in the end-of-life period, but there is limited evidence that larger-scale models can improve both utilization and the cost of care. Three models that received Health Care Innovation Awards from the Centers for Medicare & Medicaid Services aimed to improve quality of care and reduce cost through the use of innovative care coordination models. This study explores the total cost of care and selected utilization outcomes at the end-of-life for these innovative models, each of which enrolled adults with multiple chronic conditions and featured care coordination with advance care planning as a component of palliative care. These included a comprehensive at-home supportive care model for persons predicted to die within a year and two models offering advance care planning in nursing facilities and during care transitions.

**Research Design and Methods:**

We used regression models to assess model impacts on costs and utilization for high-risk Medicare beneficiaries participating in the comprehensive supportive care model (*N* = 3,339) and the two care transition models (*N* = 587 and *N* = 277) who died during the study period (2013–2016), relative to a set of matched comparison patients.

**Results:**

Comparing participants in each model who died during the study period to matched comparators, two of the three models were associated with significantly lower costs in the last 90 days of life ($2,122 and $4,606 per person), and the third model showed nonsignificant differences. Two of the three models encouraged early hospice entry in the last 30 days of life. For the comprehensive at-home supportive care model, we observed aggregate savings of nearly $19 million over the study period. One care transition model showed aggregate savings of over $500,000 during the same period. Potential drivers of these cost savings include improved patient safety, timeliness of care, and caregiver support.

**Discussion and Implications:**

Two of the three models achieved significant lower Medicare costs than a comparison group and the same two models also sustained their models beyond the Centers for Medicare & Medicaid Services award period. These findings show promise for achieving palliative care goals as part of care coordination innovation.

**Translational Significance**

Our results suggest that innovative care coordination and palliative supports can reduce end-of-life costs while improving participant and family experience. The evidence-based programs described in this paper are replicable models that could improve quality of care for aging Medicare beneficiaries across the country.

## Background and Objectives

Health systems continue to search for the most effective tools and evidence-based programs to generate value and reduce cost for their most expensive health care users. Care coordination (Ouslander, Lamb, Tappen, Herndon, & Diaz, 2011; Peikes, Chen, Schore, & Brown, 2009; Ruiz, Snyder, Rotondo, Cross-Barnet, Colligan, & Giuriceo, 2017) and palliative care supports (Cassel, Hoefer, Johnson, Kerr, & McClish, 2016; Morrison, Penrod, Cassel, Caust-Ellenbogen, & Litke, 2008) such as advance care planning, offer a potential strategy to meet those needs. Numerous observational studies have documented that palliative care is associated with reduced anxiety, fewer hospital admissions, and improved quality of life for patients and their families.(Smith, Brick, O’Hara, & Normand, 2014) Palliative care models vary but typically feature shared decision making, symptom management, and meetings with the provider and patient early in the care relationship, during which advance care planning and priorities for end-of-life are explored.(Zhang, Nilsson, & Prigerson, 2012)

The 2014 Institute on Medicine (IOM) report, *Dying in America,* argues that a majority of Americans still do not achieve their preference for dying at home and having control over their health care decisions.(Institute of Medicine, 2014) The report recommendations paint a holistic and comprehensive strategy for transformation that includes delivery of person-centered, family-oriented care, and clinician-patient communication and advance care planning. Providers looking to improve care for a diverse patient population who may or may not be approaching death raise unique questions. What changes will result in better patient experience and a reasonable return on investment? What steps can health systems take to deliver high-quality, person-centered care?

Early palliative care can result in savings in the end-of-life period (Brumley, Enguidanos, & Cherin, 2003; Colligan, Ewald, Ruiz, Spafford, Cross-Barnet, & Parashuram, 2017; Luce & Rubenfeld, 2002; Smith et al., 2003; Wallace, Walsh, Conroy, & Twomey, 2013), but there is limited evidence that larger-scale models focused on care coordination and palliative care can generate savings to offset model costs. This study explores selected end-of-life outcomes for three Centers for Medicare & Medicaid Services Health Care Innovation Award models, each of which targeted improved care and quality at the end-of-life. It is part of a larger, mixed methods evaluation of 23 Innovation Award models that served patients with multiple chronic conditions.

In 2012, Centers for Medicare & Medicaid Services’ Innovation Center awarded organizations funding to implement projects in communities across the nation that aimed to deliver better health, improved care, and lower costs to people enrolled in Medicare, Medicaid and the Children’s Health Insurance Program, particularly those with the highest health care needs. Funding for these projects was for 3 years. The objectives were to (1) engage a broad set of innovation partners to identify and test new care delivery and payment models that originate in the field and that produce better care, better health, and reduced cost through improvement for identified target populations; (2) identify new models of workforce development and deployment and related training and education that support new models either directly or through new infrastructure activities; and (3) support innovators who can rapidly deploy care improvement models (within 6 months of award) through new ventures or expansion of existing efforts to new populations of patients, in conjunction with other public and private sector partners.

The first of the three Health Care Innovation Award models included in this study is a comprehensive at-home supportive care model targeting patients predicted to die within a year. The second and third models feature the evidence-based Interventions to Reduce Acute Care Transfers (INTERACT).(Ouslander, Bonner, Herndon, & Shutes, 2014) The INTERACT toolkit provides a suite of tools to reduce transfers from skilled nursing facilities to hospitals, including advance care planning tools.

The goal of our study was to explore how these three distinct care coordination models with varying levels of intensity—each of which incorporated palliative care supports—were associated with selected end-of-life outcomes for patients. We define “high-risk” as patients with multiple chronic conditions who are usually at higher risk for hospitalization, rehospitalization, emergency department visits, or nursing home stays. We focus on total cost of care to estimate the impact (and potential cost-savings) on the Medicare program overall, rather than focusing on individual cost subcategories (e.g., costs associated with inpatient admissions). While the Affordable Care Act aimed to motivate health systems and providers in the direction of aligning medical care with patients’ needs and wishes, future incentives remain uncertain. Therefore, this article should be viewed in light of ongoing health care reform, such as reimbursement for physician advance care planning conversations and the new chronic care management code (Dresser, 2016; Szanton & Gitlin, 2016).

## Model Descriptions

Three models received Health Care Innovation Awards, with a goal of improving the quality of care and reducing utilization and cost for Medicare beneficiaries who were regarded as high-risk for readmission or death within a year through the use of care coordination and palliative or supportive care (Table 1).

**Table 1.** Summary of Innovative Care Coordination Models for High-Risk Beneficiaries

Model	Target population	Intervention	Workforce and fidelity
AIM *N* = 3,339	*Serves community dwelling older adults with prognosis of death in 12 months*	Coordinates care across multiple care settings (hospital, home health, providers’ offices, on-call triage for late-stage patients and their caregivers).	Consistent and frequent training of interdisciplinary care teams
Setting: Sutter Health System in California	35.5% of 9,406 participants were deceased at time of analysis	Supported by a unified electronic health record system and nurse-led, interdisciplinary teams.	Replication of model across sites required flexibility to fit local mix of partners and non-partners.
		Five pillars of care: (1) personal goals and advance care planning, (2) symptom management, (3) medication management, (4) follow-up with provider(s), and (5) patient engagement within the Sutter Health system.	Challenge to ensure continuity when beneficiaries are discharged from hospital given federal requirement to offer non-Sutter home health placement.
BSLTOC^a^*N* = 587	*Serves patients at assisted living and memory care units*	Model adapted INTERACT quality improvement tools in assisted living and memory care units within 48 Brookdale Senior Living sites.	Clinical and nonclinical staff received ongoing training on use of INTERACT tools.
Setting: Continuing Care Retirement Communities	39.9% of 1,473 participants were deceased at time of analysis		High turnover in residential community labor force made staff retention challenging.
IMPACT-INTERACT *N* = 277	*Serves patients being discharged from Vanderbilt hospital to skilled nursing facilities*	Two quality improvement tools—IMPACT and INTERACT—used to improve care for Medicare beneficiaries discharged from Vanderbilt University Medical Center to 23 partner skilled nursing facilities.	In-hospital discharge team led by Transitions Advocate and comprised of nurse practitioner, pharmacist, and research assistants who compile discharge plan of care and conduct warm hand-off with skilled nursing facility.
Setting: Hospital and skilled nursing facilities	31.6% of 877 participants were deceased at time of analysis		Skilled nursing facility staff trained in use of INTERACT tools

*Note*: AIM = Advanced Illness Management; BSLTOC = Brookdale Senior Living Transitions of Care.

^a^BSLTOC also serves participants in skilled nursing facilities. This analysis focuses on ambulatory care settings.

## Sutter Health Corporation’s AIM Model

The Advanced Illness Management model (AIM) targets participants with a high burden of disease and who fulfill one of three criteria: (1) meet prognosis requirements for hospice services but are not enrolled in hospice; (2) have experienced rapid or significant functional or nutritional decline, or have recurrent and unplanned hospitalizations; or (3) are considered by a physician or nurse practitioner to be likely to die in the next 12 months. The model was implemented at sites with different types of licensure requirements (home health, hospice), with organizational hosts inside and outside of the Sutter Health system. In addition to measures of utilization and Medicare costs, AIM seeks to improve the care of late-stage, medically complex patients, to reduce readmissions and enable patients to live independently in the community by providing care coordination across a continuum of settings, including home-based supports. Strategies include lengthening the duration of AIM enrollment by enrolling participants earlier in their disease trajectory before they are hospice-eligible, engaging more closely with advance care planning, and increasing the election of hospice care where appropriate.

Sutter Health has created a health information technology infrastructure to support AIM, working toward full integration with the EPIC application used in Sutter hospitals and with the Homecare Homebase electronic health record used by Sutter home health agencies. A Pillar Focused Care Note is a central communication tool of the electronic health record. While the AIM management team has emphasized model fidelity across implementation sites, it has also leveraged local staffing and partner opportunities to pilot new practices, including a clinical pharmacist consultation and referrals to an outpatient palliative care practice.

Advance care planning has been a central focus, both to familiarize patients and their families with care options, including hospice, and to facilitate related conversations across care settings and as an ongoing process. Clinicians on the AIM teams initiated and completed advance care planning, including the completion of California’s Physician Orders for Life-Sustaining Treatment form, and performance metrics included a measure of the percentage of enrollees completing an advance care plan within 90 days of enrollment. AIM patients and caregivers—both paid and family—gave high marks to the intervention, noting a greater sense of security, confidence and self-efficacy related to communication with the patient’s care team, and lowered stress on the part of caregivers (Third Annual Report: HCIA High Risk Evaluation, 2017).

## Brookdale Senior Living Transitions of Care (BSLTOC) Program

The University of North Texas, with its partner Brookdale Senior Living (BSL), has adapted the evidence-based INTERACT suite for use in assisted living and memory care settings affiliated with BSL residences in five states. The University of North Texas and Brookdale Senior Living used part of its HCIA award to pilot modified versions of INTERACT in skilled nursing facilities, home health, and independent living as well as in assisted living and memory care residences. In this article, we consider the awardee’s implementation in assisted living and memory care only. The BSL Transitions of Care (BSLTOC) model builds on a care transitions approach that BSL tested previously in 11 skilled nursing facilities across eight states. BSL also developed active data-sharing agreements with over 100 partner hospitals to actively exchange data on the discharge of BSL residents.

The BSLTOC intervention seeks to reduce transitions between assisted living or memory care settings and hospitals. The INTERACT suite includes tools and modules which improve transitions by facilitating communications among clinical and nonclinical BSL staff (associates), and between BSL and hospitals to which BSL-affiliated residents and home health clients are admitted and from which they are discharged. Model leadership emphasized advance care planning as central to the intervention, where periodic or ongoing care planning conversations with BSL residents and convening an advance care planning learning collaborative across a small group of BSL communities. In addition, INTERACT tools were enhanced to incorporate quality improvement through measurement of three key activities: (1) the frequency of advance care planning conversations with BSL residents; (2) the number of data-sharing relationships with hospitals with which BSL frequently transfers residents (high-referral hospitals); and (3) the number of weekly collaborative care meetings (convening clinicians and nonclinical residential staff) to promote fidelity to the model and allow for periodic changes to fine tune the model. Prior evaluation work on which this study is based finds that the model incorporates a number of best practices related to advance care planning, including the development of advance directives (designation of a health care surrogate and initiation of documentation), updating of patient preferences on an ongoing basis, conversion of treatment goals into portable and accessible medical orders, and making patients’ advance directives readily accessible within electronic medical records ( Third Annual Report: HCIA High Risk Evaluation, 2017).

## Vanderbilt University Medical Center’s IMPACT-INTERACT Program

Vanderbilt University Medical Center (VUMC)’s Improved Post-Acute Care Transitions and Interventions to Reduce Acute Care Transfers (IMPACT-INTERACT) model was designed to improve care and reduce readmissions for Medicare beneficiaries discharged from VUMC to one of 23 partner skilled nursing facilities in Tennessee and Kentucky. The model integrates in-hospital and postacute care services through use of a newly developed in-hospital component, known as IMPACT, which focuses on improving documentation and streamlining communication for discharge to the skilled nursing facility, and the skilled nursing facility component, known as INTERACT, which facilitates better communication among skilled nursing facility staff. The VUMC intervention seeks to reduce readmissions postdischarge from hospitals to skilled nursing facilities.

Under this model, patients admitted to VUMC are paired with a transitions advocate, generally a nurse practitioner, who works with clinical staff to conduct a series of steps while the patient is in the hospital. VUMC pharmacists work to reconcile admission, in-hospital, and discharge/transfer medication lists into a unified document that provides up-to-date information on medications the patient should be taking. The transitions advocate and research assistants within VUMC compile information from the medical record into a short summary document, known as a *nursing transition summary*, which also notes whether the patient has completed a *Physician Orders for Scope of Treatment* form or made other arrangements. During a bedside meeting with a patient prior to skilled nursing facility transfer, the transitions advocate reviews what is currently in the *Physician Orders for Scope of Treatment* form, including designations of power of attorney, do not resuscitate status, and other end-of-life care plans, or encourages the patient to complete such a form if needed. The *nursing transition summary* document is the basis for future communication between the transitions advocate and skilled nursing facility. The transitions advocate also conducts a warm hand-off call with the skilled nursing facility when the patient is discharged, reviewing the information in the *nursing transition summary* form. The transitions advocate then follows up with the skilled nursing facility after 72 hours to address any questions.

The partner skilled nursing facilities use tools from the INTERACT model, many of which are integrated into the skilled nursing facility electronic health records, that allow skilled nursing facility staff to communicate with one another in a more standardized fashion that facilitates quality improvement. Similar to BSLTOC, prior evaluation work on which this study is based finds that the model incorporates a number of best practices related to advance care planning, including the development of advance directives (designation of a health care surrogate and initiation of documentation), updating of patient preferences on an ongoing basis, and conversion of treatment goals into portable and accessible medical orders (Second Annual Report: HCIA High Risk Evaluation. 2015; and Third Annual Report: HCIA High Risk Evaluation, 2017).

However, program staff identify a substantial proportion of potentially unnecessary transfers from skilled nursing facility to hospital resulted from family members insisting that transfer be made, regardless of a patient’s documented *Physician Orders for Scope of Treatment* (CMS Quality Measures, 2017). Partner skilled nursing facility staff acknowledged the need for more time and education on end-of-life care choices, to help families of patients with advanced disease and poor prognosis to become comfortable with the patient’s expressed wishes and to serve as proxy decision makers, if necessary.

## Research Design and Methods

### Data Source and Analytic Sample

We obtained Medicare fee-for-service (FFS) claims from the Centers for Medicare & Medicaid Services Chronic Condition Data Warehouse from 2010–2016 for model participants. Claims data from 2 years prior to the study period were used to adjust our models for baseline health status (e.g., risk scores, chronic conditions). We also used the claims to identify a set of comparators matched to each participant.

Participants who were enrolled in each model between July 2013 and February 2016 and subsequently died in this timeframe were included in our study sample; these participants represented approximately 30%–40% of all model participants (Table 1). Participants included in these end-of-life analyses were comparable to nondecedents enrolled in the models, although participants at the end-of-life had, on average, higher cost and utilization (Second Annual Report: HCIA High Risk Evaluation. 2015; and Third Annual Report: HCIA High Risk Evaluation, 2017) For the BSLTOC model, the study sample included only participants in assisted living and memory care settings. We excluded participants from our analysis if they were enrolled in the model less than thirty days before death.

The comparison group in the study sample consists of individuals who died in the same time frame as participants (July 2013 to February 2016). To identify comparison groups for AIM and BSLTOC, we first identified Medicare beneficiaries living in counties similar to the models’ geographic regions based on county-level variables that include the number and characteristics of Medicare beneficiaries, Medicare Advantage penetration rate, hospice use, hospital and hospice capacities, readmission rates, emergency department visit rates, and per-capita costs. The BSLTOC comparison group was based on individuals living in similar settings (i.e., assisted living or memory care) in counties adjacent to BSLTOC within the same metropolitan area. For AIM, the comparison group was identified in neighboring California counties similar to all AIM treatment counties. For IMPACT-INTERACT, we identified patients discharged from Vanderbilt Hospital to a nonpartner skilled nursing facility. At the point of discharge, IMPACT-INTERACT participants were transferred to either a partner or nonpartner skilled nursing facility. For all three models, we used 1:1 propensity score matching to identify comparison patients most similar to model participants and who died within the same time frame. Propensity score matching models included age, gender, race/ethnicity, Hierarchical Condition Category score in year prior to program enrollment, count of hospitalizations in the last year minus the last 30 days of life, total cost of care in the last year minus the last 30 days of life, and dual eligibility (AIM and BSLTOC). Since there is limited information on disease stage and functional status in claims data, we used Hierarchical Condition Category scores and disability as indicators of overall morbidity in matching participants to comparators. The team considered using Minimum Data Set or Outcome and Assessment Information Set to adjust for factors in participants receiving skilled nursing facility or home health services. Due to data availability and sample loss, using these additional data sources was not a possibility for this analysis. For more detailed information about propensity scores and our comparison group selection and measures of quality, see the Supplementary Appendix.

### Study Design

We used a retrospective cohort study design for quantitative analysis, tracking participants and propensity-matched comparators in the 2 years prior to death, and studying differences in end-of-life outcome measures for cost and utilization for beneficiaries. We assessed differences in outcomes in the last 30, 90, and 180 days of life between model participants and the comparison group.

### Measures

We calculated five outcomes from Medicare FFS data in three domains (cost, utilization, and quality of care). Cost was measured as the total Medicare cost of care (Parts A and B services; 2013 USD) per patient in the last 30, 60, or 90 days of life; hospitalizations were measured as the number of patients per 1,000 admitted to a short-term inpatient facility in the last 30 days of life; emergency department visits were measured as the number of patients per 1,000 with an emergency department visit or a hospital observation stay (nor resulting in a hospitalization) in the last 30 days of life; and hospice care was measured as the number of patients per 1,000 who were admitted to a hospice care facility in the last 2 weeks of life. Aggregate savings to Medicare over the entire study period were obtained by multiplying the average difference in per-participant total Medicare cost of care in the last 30 days of life (as described above) by the total number of participant decedents in each model.

### Statistical Analysis

For the three Medicare cost outcomes, we used log-linked generalized linear regression models, with a gamma distribution, to obtain estimates of the average difference between the model participants and propensity-matched comparators; the same analytic sample of participants and comparators was used to compute all three outcomes. We used logit models for binary utilization outcomes. Adjusted differences were calculated as the average outcome in the intervention group minus the average outcome in the comparison group, such that a negative estimate indicates that the intervention group shows a lower rate for the outcome relative to the comparison group. We obtained robust standard errors for estimates in both cost and utilization models. All models were adjusted for demographic characteristics (age, race, gender, dual eligibility); comorbidities (Medicare eligibility due to disability, Hierarchical Condition Category risk score), (Pope, Kautter, Ellis, Ash, & Ayanian, 2004) and cost and utilization in the year prior to program enrollment. Because exposure to the model begins at enrollment in the model, we use Hierarchical Condition Category scores at time of enrollment to match participants to comparators. Conclusions drawn from these models were similar when using alternative specification (e.g., using a count outcome instead of a binary outcome). All statistical analyses were completed using Stata 13.1 and are presented with 95% confidence intervals.

## Results

### AIM

**Figure 1.**
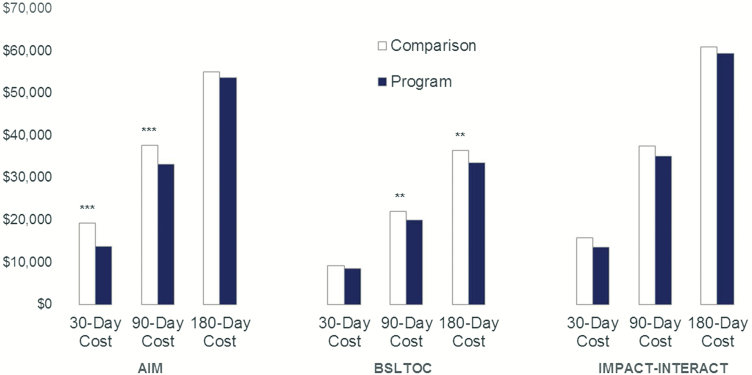
Total Medicare Cost of Care (per person) in the last 30, 90, and 180 days of life for participants in three innovative care coordination models and members of comparison groups. *Note:* Medicare claims from 2010–2016. *p < .05; **p < .01; ***p < .001. Cost assessed in the last 30, 90, and 180 days of intervention and comparison patient’s lives (AIM: 3,339 intervention patients and 3,339 matched comparison patients; BSLTOC: 587 intervention patients and 587 matched comparison patients; IMPACT-INTERACT: 277 intervention patients and 277 matched comparison patients). Lower navy blue Program bars than white Comparison bars interpreted as intervention cost savings compared to the comparison group.

AIM participants were enrolled for an average of 112 days, were approximately half female, nearly 20% were under age 65, and almost 30% were non-White participants (Table 2). We observed no significant differences in characteristics between the model participants and the matched comparison group. AIM had a significantly lower average cost per participant in the last 30 ($5,669; *p* < .001) and 90 ($4,606; *p* < .001) days of life relative to the comparison group (Table 3 and Figure 1). AIM’s 29.4% savings in the last 30 days represents a sizable reduction in Medicare cost, relative to the comparison group. For AIM, we observed a lower rate of hospitalizations (71 per 1,000 participants; *p* < .001) and a higher rate of emergency department visits relative to comparison patients (28 per 1,000 participants; *p* < .01) (Table 4). We observed that AIM participants were more likely to be in hospice in the last 14 (158 per 1,000; *p* < .001) and 30 (197 per 1,000; *p* < .001) days of life.

**Table 2.** Descriptive Characteristics of Participants in Three Care Coordination Models and Comparison Participants

	AIM		BSLTOC		IMPACT-INTERACT	
	Intervention	Comparison	Intervention	Comparison	Intervention	Comparison
	(*N* = 3,339)	(*N* = 3,339)	(*N* = 587)	(*N* = 587)	(*N* = 277)	(*N* = 277)
Mean (*SD*) Days of Enrollment	112 (150)	N/A	195 (210)	N/A	252 (252)	N/A
Maximum Days of Enrollment	1330	N/A	1123	N/A	965	N/A
Gender (%)						
Female	53.3	53.9	67.0	66.3	50.5	50.9
Age Group (%)						
<65	18.5	17.3	1.4	1.9	9.4	8.7
65‒69	11.3	10.4	1.7	2.6	10.8	8.7
70‒74	13.2	11.6	7.2	4.8	13.4	15.5
75‒79	15.1	15.6	13.1	12.9	14.4	17.7
80‒84	17.5	18.6	29.5	28.6	20.2	19.1
≥85	24.4	26.5	47.2	49.2	31.8	30.3
Race/Ethnicity (%)						
White	78.1	74.4	98.3	98.5	88.4	89.2
Black	8.9	7.5	1.2	1.2	10.5	9.7
Other	21.8	27.3	0.5	0.3	0.1	0.1
Comorbidities						
Avg. HCC Score (*SD*)	4.5 (2.2)	4.7 (2.5)	3.2 (1.8)	3.2 (1.9)	5.1 (2.5)	5.2 (2.3)
Avg. number of HCCs (*SD*)	6.4 (3.6)	6.0 (3.7)	5.0 (3.3)	5.1 (3.3)	7.9 (3.8)	8.2 (3.4)
Mean Utilization and Medicare Cost in Last Year of Life (per 1,000 beneficiaries unless noted)						
Total Cost of Care (*SD*)	$63,522 ($58,133)	$63,423 ($81,166)	$41,670 ($34,274)	$43,508 ($36,118)	$66,538 ($51,513)	$66,368 ($51,815)
Hospitalizations (*SD*)	1,776 (1,861)	1,504 (1,854)	1,063 (1,385)	1,107 (1,244)	2,274 (1,795)	2,213 (1,902)
ED Visits (*SD*)	1,368 (2,319)	1,080 (2,088)	1,165 (1,695)	1,153 (1,623)	1,498 (1,665)	1,502 (1,805)

*Note:* Source: Medicare claims from 2010–2016. There were no significant differences between intervention and comparison participants after matching. We test differences between these groups with a *t* test for continuous measures or a chi-square for categorical variables. AIM = Advanced Illness Management; BSLTOC = Brookdale Senior Living Transitions of Care; ED = Emergency department; HCC = Hierarchical condition category; SD = Standard deviation.

**Table 3.** Adjusted Difference in End-of-Life Cost Outcomes between Participants in Three Care Coordination Models and Comparison Groups

	AIM *N* = 3,339		BSLTOC *N* = 587		IMPACT-INTERACT *N* = 277	
	Estimate (95% CI)	*p*	Estimate (95% CI)	*p*	Estimate (95% CI)	*p*
Total Cost of Care (per patient)						
30-Day End-of-Life Cost	-$5,669 (-$6,602, -$4,736)***	<.001	-$861 (-$1,825, $102)^a^	.080	-$2,176 (-$4,954, $601)	.125
90-Day End-of-Life Cost	-$4,606 (-$5,990, -$3,221)***	<.001	-$2,122 (-$3,670, -$575)**	.007	-$2,422 (-$6,964, $2,121)	.296
180-Day End-of-Life Cost	-$1,348 (-$3,248, $553)	.165	-$2,922 (-$4,848, -$995)**	.003	-$1,517 (-$7,226, $4,193)	.603
Aggregate Model Savings to Medicare for Study Period^b^						
30-Day Savings	-$18,928,791 (-$22,044,078, -$15,813,504)***	<.001	-$505,407 (-$1,071,275, $59,874)	.080	-$602,752 (-$1,372,258, $166,477)	.125
90-Day Savings	-$15,379,434 (-$20,000,610, -$10,754,919)***	<.001	-$1,245,614 (-$2,154,290, -$337,525)**	.007	-$1,372,258 (-$1,929,028, -$587,517)	.296
180-Day Savings	-$4,500,972 (-$10,845,072, $1,846,467)	.165	-$1,715,214 (-$2,845,776, -$584,065)**	.003	-$420,209 (-$2,001,602, $1,161,461)	.603

*Note:* Source: Medicare claims from 2010 to 2016. AIM = Advanced Illness Management; BSLTOC = Brookdale Senior Living Transitions of Care.

**p < .01; ***p < .001.

^a^Significant at *p* < .10 level. Cost assessed in the last 30, 90, and 180 days of intervention and comparison patient’s lives (AIM: 3,339 intervention patients and 3,339 matched comparison patients; BSLTOC: 587 intervention patients and 587 matched comparison patients; IMPACT-INTERACT: 277 intervention patients and 277 matched comparison patients). Negative values interpreted as lower average cost per participant in the last 30, 90, or 180 days of life relative to a comparison group (e.g., AIM had a significantly lower average cost per participant in the last 30 ($5,669; *p* < .001) and 90 ($4,606; *p* < .001) days of life relative to the comparison group).

^b^Aggregate savings to Medicare computed by multiplying difference in average 30-day End-of-Life Medicare cost per patient by number of patients in model.

**Table 4.** Adjusted Difference in End-of-Life Utilization Outcomes between Participants in Three Innovative High-Risk Models and Comparison Groups

	AIM *N* = 3,339		BSLTOC *N* = 587		IMPACT-INTERACT *N* = 277	
	Estimate (95% CI)	*p*	Estimate (95% CI)	*p*	Estimate (95% CI)	*p*
Utilization in Last 30 Days of Life (per 1,000 patients)						
Hospice care measures	-71 (-90, -52)***	<.001	-25 (-75, 25)	.328	-45 (-125, 35)	.270
Emergency department Visits	28 (13, 43)**	.002	-2 (-41, 38)	.931	-22 (-95, 51)	.558
Hospice Care	158 (138, 178)***	<.001	34 (-19, 87)	.210	75 (20, 129)**	.007
Utilization in Last 2 Weeks of Life (per 1,000 patients)						
Hospice Care	197 (175, 219)***	<.001	27 (-24, 78)	.292	39 (-26, 105)	.241

*Note:* Source: Medicare claims from 2010–2016. **p < .01; ***p < .001. Outcomes assessed in the last 14 or 30 days of intervention and comparison patient’s lives (AIM: 3,339 intervention patients and 3,339 matched comparison patients; BSLTOC: 587 intervention patients and 587 matched comparison patients; IMPACT-INTERACT: 277 intervention patients and 277 matched comparison patients). Negative values interpreted as lower rates of hospitalizations, emergency department visits, or hospice care in the last 14 or 30 days of life relative to a comparison group (e.g., For AIM, we observed a lower rate of hospitalizations (71 per 1,000 participants; *p* < .001) and a higher rate of emergency department visits relative to comparison patients (28 per 1,000 participants; *p* < .01). We also observed that AIM participants were more likely to be in hospice in the last 14 (158 per 1,000; *p* < .001) and 30 (197 per 1,000; *p* < .001) days of life. AIM = Advanced Illness Management; BSLTOC = Brookdale Senior Living Transitions of Care.

Because Centers for Medicare & Medicaid Services invested $13 million for AIM and an almost $19 million reduction in 30-day cost was observed, there was a return on investment of $6 million for Medicare. For purposes of this analysis, we define the Centers for Medicare & Medicaid Services investment as the total amount of the award given to each model. The three models do not currently have per participant cost available to use for this analysis. The investment costs include one-time model development costs that may or may not be necessary if the model was replicated at a new site. While it is likely the cost to deliver the intervention is less than the Centers for Medicare & Medicaid Services investment, the per participant cost could be higher at sites with smaller populations of high-risk participants.

### BSLTOC

BSLTOC participants were enrolled for an average of 195 days, nearly half were 85 and older, and almost two-thirds were female (Table 2). We observed no significant differences in characteristics between the model participants and the matched comparison group. BSLTOC had a significantly lower average cost per participant in the last 90 ($2,122; *p* < .001) and 180 ($2,922; *p* < .001) days of life relative to the comparison group and a nonsignificant trend toward significantly lower average cost per participant in the last 30 days of life ($861; *p* < .10) (Table 3 and Figure 1). We did not observe statistically significant lower rates of hospitalizations or emergency department visits in the last 30 days of life for BSLTOC (Table 3). We also observed that BSLTOC participants were no more likely to be in hospice in the last 14 or 30 days of life.

### IMPACT-INTERACT

IMPACT-INTERACT participants were enrolled for an average of 252 days, were half female, approximately two-thirds were 75 and older, and roughly 10% were non-White participants (Table 2). We observed no significant differences in characteristics between the model participants and the matched comparison group. We did not observe any significant cost savings in the last 30, 90, or 180 days of life for IMPACT-INTERACT participants relative to the comparison group (Table 3 and Figure 1). While we did not observe statistically significant lower rates of hospitalizations or emergency department visits in the last 30 days of life for IMPACT-INTERACT, we did observe that IMPACT-INTERACT participants were more likely to be in hospice in the last 30 days of life (75 per 1,000; *p* < .01) (Table 4).

## Discussion and Implications

This study suggests that models with carefully planned care coordination and that include deliberate advance care planning have the potential to transform end-of-life care in the United States and can have favorable outcomes for payers concerned with rising costs and unnecessary health care utilization. We found that two models which focused on care coordination for high-risk beneficiaries were associated with reductions in measures of end-of-life costs, relative to matched comparison groups. The observed higher rates of emergency department utilization for AIM participants may be due to AIM specifically targeting medically frail patients with recurrent, unplanned health care utilization and twice the average number of comorbidities compared to controls. A common component among the three models was use of advance care planning and early end-of-life conversations.

Duration of intervention and setting play an important role in whether models are able to realize large cost savings. AIM was able to target beneficiaries in the community for up to a year or more prior to death, which may have contributed to better cost outcomes. BSLTOC participants also may have benefited from being part of their continuing care retirement community (CCRC) long before their terminal decline, given the intervention’s focus on periodic advance care planning conversations. IMPACT-INTERACT participants, on the other hand, had the shortest exposure to the intervention and achieved nonsignificant savings, though their study sample was likely underpowered to detect differences. Because participants were enrolled in the IMPACT-INTERACT model at such a late stage of their end-of-life trajectory, it may have been too late to make a significant impact.

We would be remiss if we did not consider alternative positions about the value and impact of these models. First, it is possible that each model may result in improvements in quality of life for patients and families unmeasured in this analysis, but the costs associated with the intervention may be more than usual care for some providers making them less likely to be sustained. Each practice will need to weigh the cost of integrating innovation into their work flow against the benefits of participant satisfaction and improved quality of care. As the Centers for Medicare & Medicaid Services continues its march toward quality and linking payment to quality, further alignment of incentives will be essential for this shift to occur.(CMS Quality Measures, 2017). Additionally, we cannot expect that every Centers for Medicare & Medicaid Services model will reduce end-of-life cost through care coordination and palliative care. In our study, we find that some innovations such as IMPACT-INTERACT were not able to demonstrate cost savings during the study period. Other interventions, such as AIM and BSLTOC, that were successful in reducing costs overall, were not uniformly effective at reducing utilization. Further, some utilization, such as emergency department visits at the end of life, may actually increase. Placing these results in a broader context, while there are a number of factors that can influence cost outcomes for palliative care services such as disease(s), age group, or care setting, this study supports findings elsewhere that palliative care services can be highly effective at reducing costs at the end of life (Smith et al., 2014). These results also suggest that for some high-risk patients, high costs may not be reducible, especially in their last days.

However, as noted above, family member interactions sometimes worked against model goals of reduced readmissions. BSLTOC and IMPACT-INTERACT both struggled to reconcile patient preferences to remain in the community and family preferences of wanting their loved one to return to the hospital. BSLTOC staff described being confident and prepared to treat residents on site, while family members pressed for emergency department visits based on their perception that more could be provided at the hospital. IMPACT-INTERACT leadership noted that families pushed for return to the hospital rather than return to the community during skilled nursing facility discharge out of concern that suitable care could not be provided at home. These observations suggest that a longer window is needed to secure family buy-in concerning end-of-life planning and choices.

Our findings both confirm previous research and introduce valuable new findings to the literature on care coordination and end-of-life cost savings. A unique contribution of this study is the proof that even at varying levels of exposure (e.g., 1 year vs a few months prior), care coordination and palliative supports can lower end-of-life costs and utilization. To date, most literature has focused on highly specialized patient populations, unlike the diverse beneficiaries with multiple chronic conditions served by the models in this paper. This suggests that diverse populations can benefit from evidence-based care coordination.

The following limitations of our analyses should be noted. First, while we tailored the comparison group for each model based on the participants served by the model, estimates across the three models are not directly comparable since the models served different participants based on model setting and patient need. Second, because we used Medicare claims as the primary data source for the quantitative analyses, we were only able to use covariates observed in claims. Unobserved differences between the model participants and comparison groups may persist, even after rigorously matching the two groups on observed characteristics. Additionally, the factors with which we propensity-matched our treatment group to the comparison group for BSLTOC and IMPACT-INTERACT included data within the last 90 and 180 days of life; thus, our longer-term outcomes may be affected by this endogeneity. Finally, the external generalizability of our findings may be limited. The models we studied applied for a Health Care Innovation Award, which suggests that compared to typical providers, they may be particularly motivated to improve care and reduce cost (Skillman et al., 2017).

These findings are particularly important in light of ongoing efforts to provide value in health care through alternative payment models. Rather than create new programs, health systems should consider use of care transition programs such as INTERACT to improve end-of-life outcomes. Two of the three models (AIM and BSLTOC) are sustaining their innovations in whole and routinely provide consultant services to organizations (i.e., health systems or continuing care retirement communities) interested in implementing similar programs. Sutter Health is investing its own funds to underwrite the components that are not eligible for Medicare reimbursement and pursing opportunities to contract with payers. BSLTOC has integrated INTERACT protocols into their electronic health records and plans to maintain data exchange with partner hospitals in most markets. IMPACT-INTERACT is not being sustained, though components are expected to be integrated into existing hospital operations.

Collectively, the findings present a strong business case for integrating these components into care coordination models. The Centers for Medicare & Medicaid Services investment in AIM resulted in a return on investment of $6 million for Medicare. This finding, combined with the quantitative findings for the AIM and BSLTOC models and the qualitative findings for all three models, suggests that a carefully planned care coordination intervention with deliberate advance care planning has the potential to improve end-of-life experience and reduce costs and utilization.

## Supplementary Material

Supplementary data is available at *Innovation in Aging* online.

## Funding

The work was supported by the Centers for Medicare and Medicaid Innovation (Contract No. HHSM-500-2011-00002I, Order No. HHSM-500-T00010).

## Conflict of Interest

None reported.

## Supplementary Material

igx021_suppl_Appendix
