# Supplementary material for: Innovative Models for High-Risk Patients Use Care Coordination and Palliative Supports to Reduce End-of-life Utilization and Spending
Source: Innov Aging. 2017 Nov 20;1(2):igx021. doi: 10.1093/geroni/igx021 (PMC6176974; doi:10.1093/geroni/igx021)
Supplement: igx021_suppl_Appendix [file igx021_suppl_appendix.docx]

**Appendix**

Part I: Quantitative Methods

1. **Data Source**

Our data sources include the Medicare beneficiary enrollment database and Medicare fee-for-service claims (Part A and Part B) located on the Chronic Condition Warehouse (CCW) Virtual Research Data Center (VRDC) data enclave environment. We also used files obtained from each model that identify participants and their enrollment dates. We linked these files to Medicare claims, and created a longitudinal person-level analytic file for each participant for each of the models (AIM, BSLTOC, IMPACT-INTERACT). Each participant-level observation contained a summary of cost, utilization, and quality measures pertaining to the last year of life for that participant. Medicare data on the CCW VRDC were also used to create the comparison groups.

1. **Measure Specification**

We calculated five core measures in three domains (cost, utilization, and quality of care) for each of the models, as seen in Exhibit A1.

Exhibit A1: Measure Specification

| Domain | Measure | Definition |
| --- | --- | --- |
| **Total Cost of Care** | 30-Day Cost | Total Medicare cost of care (Parts A and B services) per patient in the last 30 days of life |
|  | 90-Day Cost | Total Medicare cost of care (Parts A and B services) per patient in the last 90 days of life |
|  | 180-Day Cost | Total Medicare cost of care (Parts A and B services) per patient in the last 180 days of life |
| **Utilization** | Hospitalizations | Number of patients per 1,000 admitted to a short-term inpatient facility in the last 30 days of life |
|  | ED Visits | Number of patients per 1,000 with an ED visit or a hospital observation stay (not resulting in hospitalization) in the last 30 days of life |
| **Quality of Care** | Hospice Care | Number of patients per 1,000 who were admitted to a hospice care facility in the last two weeks of life |

*Note.* Total cost of care is expressed in 2013 dollars after adjusting for medical care consumer price index.

1. **Dataset Construction**

To construct analytic files, we began with claims-level data and identified participants using unique patient identification numbers, selecting all claims for patients enrolled at one of the models during the model timeframe. We included participants who had been enrolled in the model for 30 or more days. In addition to outcome measures presented in the manuscript, each analytic file included:

- Centers for Medicare and Medicaid Services (CMS) region, state, county, and zip code of residence;
- available patient demographics: age, gender, race/ethnicity, dual eligibility, and reason for Medicare eligibility (e.g., age, disability, end stage renal disease);
- CMS’s Hierarchical Condition Category (HCC) risk score or Charlson Comorbidity Scores for the 12 months before enrollment in the model;(1)
- chronic and acute condition flags, identified by Clinical Classifications Software (CCS) codes; and
- utilization of hospital and outpatient emergency department care for the 12 months before enrollment in the model.

1. **Comparison Group Selection**

We included a comparison group for all three models in this manuscript. For each model, we used a three-stage process to define the comparison group:

1. ***Identify comparison sites***

First, we selected ***sites*** comparable to the model implementation site. Comparison ***sites*** are listed for the three models are described in Exhibit A2.

For AIM, we identify neighboring counties for all AIM treatment counties based on a set of county-level variables that include the number and characteristics of Medicare beneficiaries, Medicare Advantage penetration rate, hospice use, hospital and hospice capacities, readmission rates, ED visit rates, and per capita costs. We then used propensity scores to identify the comparison beneficiaries in adjacent geographic regions based on patient demographics, disability eligibility, HCC scores; and number of hospitalizations, ED visits, and total cost of care in the past 60 days.

For BSLTOC, we identify neighboring counties for all BSLTOC treatment counties by comparing on key characteristics from the Health Resources and Services Administration (HRSA) county-level area resource file (ARF) including: count of FFS beneficiaries, percent non-Hispanic White, standard risk adjusted per-capita cost, ED-visits per-1000, average age, and HCC score.^[[1]](#footnote-1)^ We then include Medicare beneficiaries who live in assisted living residences located in counties similar and adjacent to counties with BSLTOC assisted living residences.

For IMPACT-INTERACT, we include participants randomly discharged to non-partner SNFs from VUMC, regardless of SNF site or geographic location.

Exhibit A2: Sampling Frame

| Model | Sampling Frame | Comparison Areas |
| --- | --- | --- |
| **AIM** | Medicare beneficiaries who live in similar geographic areas and who died during 2013-2015 | **California:** Alameda County, Santa Clara County |
| **BSLTOC** | Medicare beneficiaries who had any FFS claim with a Place of Service code flagged as an AL residence located in an adjacent county to a BSLTOC AL residence, and who died during the program timeframe | **Texas:** Collin County, Montgomery County, Williamson County, Denton County, Galveston County, Fort Bend County, Brazoria County  **Florida:** St. John’s County |
| **IMPACT-INTERACT** | Medicare beneficiaries discharged immediately after hospitalizations from VUMC into a non-participating SNF, and who subsequently died during the program timeframe | All beneficiaries discharged from VUMC to non-participating SNFs, regardless of site or geographic area |

*Note.* Both the AIM model and the BSLTOC model had multiple model sites; we identified comparison practices for each site separately.

1. ***Limit to qualified patients***

Next, we select all beneficiaries residing in the selected geographic area or receiving treatment from the selected comparison practices. We identify these beneficiaries using Medicare enrollment and claims data. We select all beneficiaries who enroll prior to 2015 and meet the awardee-specific inclusion and exclusion criteria.

1. ***Select similar patients***

Finally, we used propensity score methods to match participants to comparators with similar characteristics. We estimated the propensity score using logistic regression as the probability of a patient being enrolled in each model, conditional on the patient’s covariates. Exhibit A3 summarizes the approach to propensity score models and the variables used for each model.

Variables in the propensity score model included, but were not limited to, patient demographics, clinical covariates, morbidity, prior utilization, and characteristics of the provider/area. **T_i_** is the probability of being a treatment group, **Patient_i_** is a vector of patient characteristics, and **Practice/Area_i_** is a vector of characteristics of the practice or the area for the participant. The following specification was used for the propensity score models:

Logit[Pr(T_i_=1)] = β_0_ +β_1_Patient_i_ +β_2_Practice/Area_i_

We assessed and confirmed both common support as well as covariate balance between the treatment and comparison group patients before and after applying propensity scores. We then compared the two groups—treated and comparison—to estimate the effects of the intervention.

Exhibit A3: Variables Used in Propensity Score Models

| Model | Variables |
| --- | --- |
| **AIM** | Age (continuous); gender; race/ethnicity; dual eligibility; HCC score in year prior to model enrollment; count of hospitalizations in the last year minus the last 30 days of life; total cost of care in the last year minus the last 30 days of life. |
| **BSLTOC** | Age (continuous); gender; race/ethnicity; dual eligibility; HCC score in year prior to model enrollment; count of hospitalizations in the last year minus the last 30 days of life; total cost of care in the last year minus the last 30 days of life. |
| **IMPACT-INTERACT** | Age (continuous); gender; race/ethnicity; HCC score in year prior to model enrollment; count of hospitalizations in the last year minus the last 30 days of life; total cost of care in the last year minus the last 30 days of life. |

1. **Analytic Approach**

For measuring each model’s effect on our outcome measures, we calculated the difference in outcomes (e.g., cost in the last 30 days of life) between the treatment and matched comparison group using generalized linear regression models. The model specifications are presented in Exhibit A4. We also computed the percentage difference between the treatment and comparison group.

Exhibit A4: Regression Model Specifications

| Domain | Regression Model | Distribution | Stata Command |
| --- | --- | --- | --- |
| **Cost** | Generalized linear regression model with a log link | Gamma | xtgee |
| **Utilization** | Generalized linear regression model with a log link and robust standard errors for counts of utilization | Negative Binomial | glm |
| **Quality of Care** | Logistic regression model with a log link and robust standard errors for binary indicators or quality | Logit | logit |

Part II: Supplemental Results

1. **Propensity Score Matching**

Exhibits A5 and A6 summarize the results from our propensity score-based comparison group selection for the AIM, BSLTOC, and IMPACT-INTERACT models. Exhibit A6 shows the similarities between the treatment and comparison groups before and after propensity score matching. After matching, we observed that the treatment and comparison groups for all three models have nearly identical distributions of propensity scores.

Exhibit A5: Common Support for BSLTOC, AIM, and IMPACT-INTERACT Participants and Comparison Participants

1. **AIM**


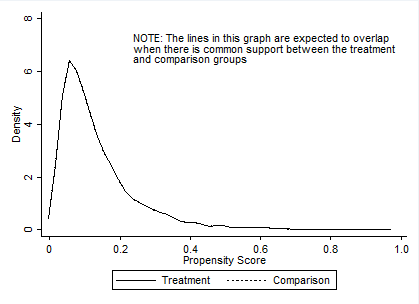


1. **BSLTOC**


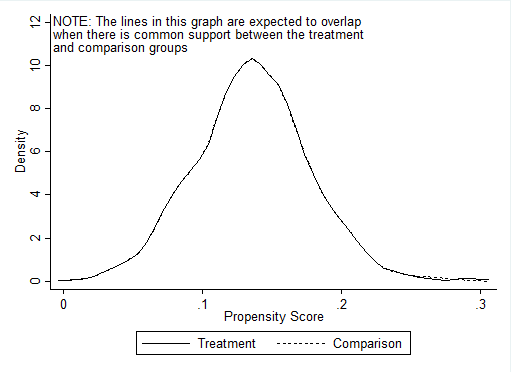


1. **IMPACT-INTERACT**


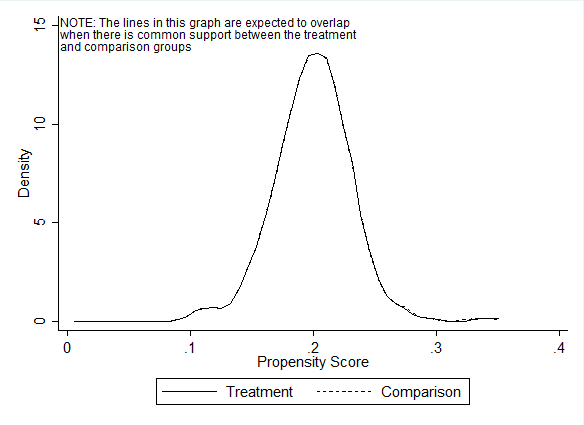


Exhibit A7 shows the distribution of covariates before and after matching for each of the three models; the vertical lines on the graph indicate the accepted outer bounds of covariate difference for propensity score matching (standardized difference in means between -10% and 10%). These charts indicate that no variables showed greater than 10% standardized difference in means between the intervention and matched comparison beneficiaries. Thus, propensity score matching greatly improved the comparability of the treatment and comparison groups.

Exhibit A7: Covariate Balance for BSLTOC, AIM, and IMPACT-INTERACT Participants and Comparison Participants

1. **AIM**


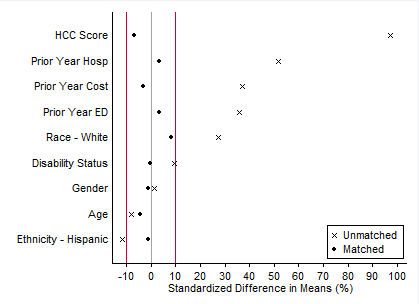


1. **BSLTOC**


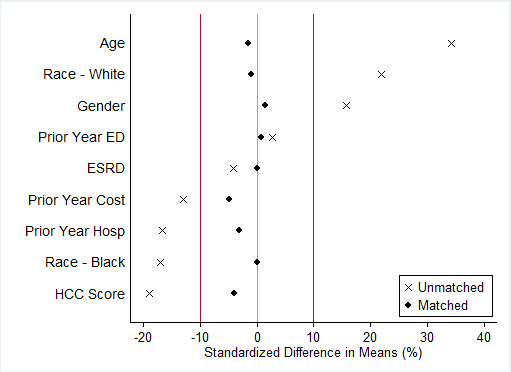


1. **IMPACT-INTERACT**


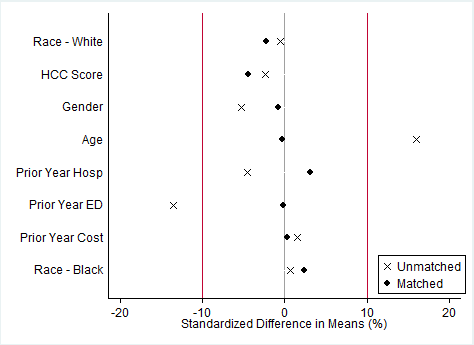


1. **Cost, Utilization, and Quality of Care**

Exhibits A7-A8 present the overall averages for the treatment and comparison groups, as well as the difference between the treatment and comparison groups, for each of the three models for cost, utilization, and quality of care outcomes, respectively.

Exhibit A7: Average Cost Outcomes for BSLTOC, AIM, and IMPACT-INTERACT Participants and Comparators

|  | 30-Day Cost | 90-Day Cost | 180-Day Cost |
| --- | --- | --- | --- |
| **AIM** | | | |
| Treatment | $13,623 | $33,063 | $53,695 |
| Comparison | $19,292 | $37,668 | $55,043 |
| Difference | -$5,669 | -$4,606 | -$1,348 |
| % Difference | -29.4 | -12.2 | -2.4 |
| **BSLTOC** | | | |
| Treatment | $8,364 | $19,906 | $33,527 |
| Comparison | $9,225 | $22,028 | $36,449 |
| Difference | -$861 | -$2,122 | -$2,922 |
| % Difference | -9.3 | -9.6 | -8.0 |
| **IMPACT-INTERACT** | | | |
| Treatment | $13,555 | $35,051 | $59,361 |
| Comparison | $15,732 | $37,473 | $60,877 |
| Difference | -$2,176 | -$2,422 | -$1,517 |
| % Difference | -13.8 | -6.5 | -2.5 |

Exhibit A8: Average Utilization Outcomes for BSLTOC, AIM, and IMPACT-INTERACT Participants and Comparators (per 1,000)

|  | Hospitalizations | ED Visits | Hospice |
| --- | --- | --- | --- |
| **AIM** | | |  |
| Treatment | 418 | 175 | 319 |
| Comparison | 489 | 147 | 161 |
| Difference | -71 | 28 | 158 |
| % Difference | -14.5 | 19.0 | 98.1 |
| **BSLTOC** | | |  |
| Treatment | 351 | 147 | 495 |
| Comparison | 376 | 148 | 461 |
| Difference | -25 | -2 | 34 |
| % Difference | -6.7 | -1.2 | 7.4 |
| **IMPACT-INTERACT** | | |  |
| Treatment | 508 | 261 | 218 |
| Comparison | 553 | 283 | 179 |
| Difference | -45 | -22 | 39 |
| % Difference | -8.1 | -7.8 | 21.9 |

1. <http://ahrf.hrsa.gov/download.htm> . [↑](#footnote-ref-1)
